# Methods of estimation of mitral valve regurgitation for the cardiac surgeon

**DOI:** 10.1186/1749-8090-4-34

**Published:** 2009-07-15

**Authors:** Efstratios E Apostolakis, Nikolaos G Baikoussis

**Affiliations:** 1 Cardio-Thoracic Surgery Department, School of Medicine, University Hospital of Patras, Patras, Greece

## Abstract

Mitral valve regurgitation is a relatively common and important heart valve lesion in clinical practice and adequate assessment is fundamental to decision on management, repair or replacement. Disease localised to the posterior mitral valve leaflet or focal involvement of the anterior mitral valve leaflet is most amenable to mitral valve repair, whereas patients with extensive involvement of the anterior leaflet or incomplete closure of the valve are more suitable for valve replacement. Echocardiography is the recognized investigation of choice for heart valve disease evaluation and assessment. However, the technique is depended on operator experience and on patient's hemodynamic profile, and may not always give optimal diagnostic views of mitral valve dysfunction. Cardiac catheterization is related to common complications of an interventional procedure and needs a hemodynamic laboratory. Cardiac magnetic resonance (MRI) seems to be a useful tool which gives details about mitral valve anatomy, precise point of valve damage, as well as the quantity of regurgitation. Finally, despite of its higher cost, cardiac MRI using cine images with optimized spatial and temporal resolution can also resolve mitral valve leaflet structural motion, and can reliably estimate the grade of regurgitation.

## Introduction

The classical indications for surgical intervention of patients with mitral regurgitation are based either on the symptoms, or on the function of left ventricle and the estimated degree of regurgitation in the non-symptomatic patients [1]. According to the 2007 guidelines of American Heart Association [2] mitral valve (MV) surgery is recommended: 1. for symptomatic patients with acute severe mitral regurgitation (MR). 2. MV surgery is beneficial for patients with chronic severe MR and NYHA functional class II, III, or IV symptoms in the absence of severe left ventricle (LV) dysfunction (severe LV dysfunction is defined as ejection fraction less than 0.30) and/or end-systolic dimension greater than 55 mm. 3. MV surgery is beneficial for asymptomatic patients with chronic severe MR and mild to moderate LV dysfunction, ejection fraction 0.30 to 0.60, and/or end-systolic dimension greater than or equal to 40 mm. 4. MV repair is recommended over MV replacement in the majority of patients with severe chronic MR who require surgery, and patients should be referred to surgical centers experienced in MV repair [2]. In the cases of ischemic mitral regurgitation, the decision to operate the mitral valve in combination with bypass grafting is more difficult, and should generally be made preoperatively. According to the ACC/AHA guidelines, it is indicated if the severity of regurgitation is characterized "severe", namely 3+ or 4+, and also a significant left ventricular dysfunction is evident [2]. Bolling S. reported in his article that a vicious cycle of continuing volume overload, ventricular dilation, progression of annular dilation, increased LV wall tension and worsening MR and heart failure occur [3]. In other words, in every case of mitral regurgitation, the indication for surgical intervention is based on a reliable quantification of several paraclinical methods. How reliable are these methods? Three are the methods of preoperative estimation of mitral regurgitation: cardiac catheterization, Doppler echocardiography and magnetic resonance imaging (MRI). We would like to compare these diagnostic methods and the information which provide each of them to cardiac surgeon.

### A) Cardiac catheterization

Valvular regurgitation can be evaluated by angiography. Angiographic evaluation of regurgitant severity is based on ejection of contrast media into the left atrium, through the affected mitral valve, or into the left ventricle through the insufficient aortic valve [1]. The severity of regurgitation is graded on a semi quantitative scale of 0+ to 4+ (see table 1).

**Table 1:** Angiographic grading of regurgitant severity of aortic and mitral valve [1].

**Grade**	**Aortic regurgitation**	**Mitral regurgitation**
1+	Contrast refluxes from the aortic root into the left ventricle but clears on each beat	Contrast refluxes into the left atrium but clears on each beat

2+	Contrast refluxes into the left ventricle with a **gradually increasing density **of contrast in the left ventricle that never equals contrast intensity in the aortic root	Left atrial contrast density gradually increases but **never equals **left ventricle density

3+	Contrast refluxes into the left ventricle with a **gradually increasing density **such that left ventricle and aortic root **density are equal **after **several **beats	The density of contrast in the atrium and ventricle equalize after **several **beats

4+	Contrast fills the left ventricle resulting in an **equivalent radiographic density **in the left ventricle and aortic root on the first beat	The left atrium becomes **as dense as **the left ventricle on the first beat and contrast is seen refluxing into the pulmonary veins

The points of obscureness are in bold or with questionmarks.

#### Severity of mitral valve regurgitation

It is evident from table 1, that the distinction between the 4 several grades of regurgitation are difficult and in most cases inaccurate. This mode of estimation of degree of regurgitation has some important limitations, which confutes its usefulness: a) the quantity of contrast material (volume and speed of injection) is proportional of density and if this is small may downregulate the grade of regurgitation [4,5], b) the arrhythmia (ventricular extra-beats or atrial fibrillation, or even that produced by the catheter itself) significantly affects the ventricular filling and subsequently the indicated grade of regurgitation[5], c) although mild regurgitation is clearly distinct from severe regurgitation, intermediates grades may not be reliable estimated [1], d) the position of catheter in the ventricle (for mitral valve) or in the aorta (for aortic valve), in relation to the site of valve[5], e) the recorded plane of ventricle and/or atrium, to avoid overlapping. The "ideal" plane for estimation of aortic regurgitation is that of 45° in left anterior oblique view with 10–15% of cranial angulation, while that for mitral regurgitation is a 30° in right anterior oblique view [1]. f) avoid the overlapping of descending thoracic aorta and left atrium which may overestimate the mitral regurgitation [1], g) avoid derangements of preload and afterload (systemic and pulmonary vascular resistance for aortic and mitral valve, respectively) which significantly affects the grade of regurgitation, h) the coexistence of mitral and aortic regurgitation can change the regurgitant contrast volume through the mitral valve and therefore overestimates the grade of its regurgitation [6]. According to Otto C [6], angiography offers evaluation of grade of regurgitation only "in selected cases", and especially when the non-invasive evaluation is inconsonant to the clinical findings. The advantages and disadvantages of angiography, Doppler and MRI are presented in table 2.

**Table 2:** Advantages and disadvantages of three methods of estimation of left-sided valve's regurgitation.

**Mode of evaluation**	**Advantages**	**Disadvantages**
**Angiography**	-simultaneous calculation of SVR, PVR, LVEDP, PCWP, EF, etc ^(1,6)^ -easy interpretation by cardiologists and cardiac surgeons ^(6)^	-invasive method, risk of complications^(5)^ -misinterpretation in double valve disease ^(1,6)^ -higher cost ^(5,6)^ -temporarily affects hemodynamic of patient (SVR, PVR) and obscure the results ^(5)^ -time-consuming

**Doppler**	- non-invasive method - no risks - low-cost - time-consuming - does not affect hemodynamically the patient - semi-quantitative	- overlapping structures - "bad" window - operator depending discomfort+hemodynamic interaction of TEE – limitations (see text) - influence of site of Jet

**MRI**	- measurement of LVEDV, LVESV, LV mass - no risk - non invasive tool - precise and valid estimation ^(23,24)^ - does not need suppression or anaesthesia (such as TEE) - estimation of myocardial function and viability ^(9,29)^	- respiratory interference -not-hemodynamic measurement - not-anatomic information - discomfort for the patient - artefacts in the case of metallic materials ^(40,41)^

(SVR: systemic venous resistance, PVR: pulmonary venous resistance, PCWP: pulmonary capillary wedge pressure, EF: enjection fraction, LVEDV: left ventricle end diastolic volume), LVESV: left ventricle end systolic volume, LV mass: left ventricle mass, TEE: Transesophageal echocardiography).

#### Advantages and disadvantages of angiography, Doppler and MRI

Another significant component of estimation of valve regurgitation is the calculation of regurgitant volume and fraction. Regurgitant volume can be calculated by the formula: regurgitant SV = total SV-forward SV, where total SV is the total amount of ejected blood by the LV, measured from left ventricular angiogram, and forward SV the amount of blood ejected through the aortic valve, and measured by Fick's thermodilution technique [6]. According to this equation, may be measured the regurgitation fraction which characterizes the severity of valve regurgitation: for < 20% mild, 20–40% moderate, 40–60% moderately severe, and > 60% severe regurgitation [5]. Unfortunately, this method also has its limitations. First, its accuracy is depended on the accuracy of measurements of cardiac output [6], and second, in the case of coexistence aortic and mitral regurgitation, only a rough estimation of the portion of regurgitant fraction of each valve can be made [6].

### B) Doppler Echocardiography

Mitral regurgitation is a relatively common and important heart valve lesion in clinical practice and adequate assessment is fundamental to decisions on management. Echocardiography is the recognized investigation of choice for mitral valve regurgitation [7,8]. However, the technique is operator dependent and may not always give reliable diagnostic views for estimation of mitral valve dysfunction. Transesophageal echocardiography, with 3-dimentional visualization if available, generally gives a better overall assessment of mitral valve dysfunction and the lesions responsible for it, but is also operator dependent, semi-invasive and usually requires patient's sedation [9]. The latter may affect the quantity of mitral regurgitation especially in the cases of ischemic origin. According to Aklog L [10], 90% of their patients with moderate (3+/4+) mitral regurgitation who underwent intraoperative TEE had their MR downgraded to mild or less (1+-2+/4+), and in 30% of their patients, there was no detectable MR on intraoperative TEE. Similar results reported and other studies concerning the influence of anaesthesia and sedation on downgrading of real mitral regurgiration [11,12]. It is recognised that TEE assesses the mechanisms of valve dysfunction well (leaflet prolapse/restriction) and is perhaps the technique best able to determine the structural lesion responsible for the incompetence (chordal/papillary muscle rupture/elongation, leaflet perforation, etc). Although TTE images using harmonic imaging can usually identify leaflet abnormalities in mitral valve prolapse, many patients will have poor image quality due to, reduced ultrasound penetration through scar tissue, air filled lung or excess adipose tissue [13]. Because of variation in image quality and imaging widows systematic segmental mapping of the mitral valve leaflets is often not attempted using 2-dimensional TTE in clinical practice. The standard echocardiographic examination generally in a valvular disease is based on Doppler colour flow imaging, on pulsed Doppler transvalvular velocities, and on continuous wave Doppler measures of regurgitant severity [14]. Doppler colour flow imaging is used to estimate the severity of aortic or mitral valve regurgitation. The amount of regurgitant jet within the antecedent chamber (namely LV for the aortic regurgitation and left atrium for the mitral regurgitation), is directly proportional to the severity of valve regurgitation [15]. However, there are also included some important limitations in this method: a) a small degree of mitral and aortic valve regurgitation is seen in 70–80%, and in 5–10% respectively, of normal individuals [14], b) typically the size of the jet is indexed to the size of the left atrium, and it is a drawback of this method for precise estimation of mitral regurgitation [15]. C) The site of jet affects the measured grade of regurgitation. Jets that are peripheral or impinging on a wall, rather than centrally, cause underestimation of severity of regurgitation (of regurgitant volume) up to 40% [16,17]. Quantification of mitral regurgitation is also heavily dependent on the colour Doppler flow area, and pertains to the holosystolic regurgitation [15]. The criteria of characterization of severity of mitral regurgitation are included in the table 3.

**Table 3:** The severity of mitral regurgitation according to the Doppler echocardiography ([15]

**Severity Of Mitral Valve Regurgitation**	**I (mild)**	**II**	**III**	**IV (severe)**
**Jet (% Left atrium)**	< 15%	15–30%	35–50%	> 50%

**Spectral Doppler**	**Faint**	-	-	**Dense**

**Vena contracta**	< 3 mm	-	-	> 6 mm

**Pulmonary vein flow**	S > D	-	-	Systolic reversed

**Right ventricle (ml)**	< 30	30–45	45–59	> 60

**Effective regurgitant orifice (ERO in cm2)**	< 0.20	0.20–0.29	0.30–0.39	> 40

**Proximal Isovelosity Surface Area (PISA)**	**small**	-	-	**large**

The points of inaccuracy are depicted in bold or with question marks.

#### Criteria of characterization of severity of mitral regurgitation

It is obvious from table 3: a) that the distinction between mild and severe grade of regurgitation by using the method is easy, but not for the intermediate (II and III) grades, and b) the measurement all of above mentioned parameters of Doppler ECHO is dependent on many others hemodynamic paramenters such as the preload, afterload, and rhythm [14], anatomic parameters such as the dimensions of left atrium [15], or on technical parameters such the "window" [14], as well as on other parameters such as operator's experience, ability of device, etc. For these reasons, interpretation of colour flow data is quite variable, with a disagreement of 29% for aortic and 25% for mitral regurgitation [14,18].

There are also some others proposed semi-quantitave mitral regurgitation indices, such as a scoring system of severity, with included most of parameters of table 3, and scored each of them between 0 and 3 [17,19]. According to this system, the total score is divided through the number of evaluated parameters, and for index > 2.2 indicates a severe regurgitation, > 1.7 a mild, and index from 1.7 to 2.2 indicate an intermediate or better, vague estimation of regurgitation. However, and this method has the same limitations which reported earlier, plus that of values approximation (methodological problems).

According to Loick et al [20], the intraoperative echocardiographic assessment of mitral regurgitation is reliable, simple and relatively unaffected by hemodynamics. It means that in the one side, it may be involved fewer imponderable factors, but on the other side, it may not be acceptable from a surgical point of view, because it may underestimate the grade of regurgitation [14,20,21].

The regurgitant volume is estimated by using the proximal isovelocity surface area (PISA) on colour Doppler imaging [22,23]. This measurement has also several important limitations such as the integration of diastolic flow, as well as caveats of used mathematic types [14].

### C) Magnetic Resonance Imaging

Magnetic Resonance Imaging is a third method for estimation (quantification) of mitral valve regurgitation. The assessment is based on estimation of regurgitant volume by determining the difference between the stroke volumes of ventricles [24]. Stroke volumes are calculated from a stack frame of images as the difference between and-diastolic and end-systolic volumes for each ventricle [24]. In the normal individuals, stroke volume of right ventricle is nearly equivalent to that of left ventricle. Every difference between the two measured stroke volumes indicates the amount of blood which comes back through the insufficient valve during diastole. The estimation is precise, but the limitations of method are the following: a) the measurement is reliable only for the case of single regurgitant valve; in the cases of combined aortic and mitral regurgitation, the difference represents the sum of regurgitant volume [23]. b) The estimation is valid, only if the tricuspid valve is sufficient [23,24]. Another method for quantification of valve regurgitation is the cine MRI [24,25]. Especially for the aortic valve, this method can discriminate between antegrade and retrograde flow during the cardiac cycle by analysis of bright or dark voxels in the ascending aorta, enabling retrograde flow to be directly measured [24,26]. Diastolic retrograde aortic flow equals aortic regurgitant volume, and correlates closely with volumetric cine MRI [23,24].

According to Kizilbash et al [27], the MRI provides accurate measurements of regurgitant flow that correlate well with quantitative Doppler imaging, and in addition, it is the most accurate non-invasive technique for measuring ventricular end-diastolic volume, end-systolic volume and left ventricular mass. Concerning the estimation of mitral regurgitation MRI in the last years is considered more reliable in comparison to these of echocardiography. In fact, there are many studies comparing the two methods [7,8]. Cardiovascular MRI has many advantages like accurate determination of left as well as right ventricular volumes and function [28,29], measurements of aortic flow volume, and in ischemic mitral regurgitation, comprehensive assessment of regional myocardial function and viability [28,30]. When used optimally, MRI can complement echocardiography in the assessment of mitral regurgitation, especially in patients in whom transthoracic echocardiography has not provided adequate information [9]. Finally, MRI has been proved that overtake the limitations of ecocardiography (overlapping structures, "bad window", artefacts, or contraindications of TEE. On the other hand, in its limitations are included the respiration, and the higher cost [7].

The mitral regurgitant volume (MRV) measured by MRI is the difference between the LV stroke volume (LVSV) and the aortic forward stroke volume (AoSV) i.e. MRV (mls/beat) = LVSV – AoSV. The regurgitant fraction (RF) is the ratio of the MRV divided by the LVSV i.e. RF (%) = (MRV × LVSV) × 100 [9].

It may also be possible to directly measure mitral inflow volume by phase-contrast velocity flow mapping at the tips of the mitral valve leaflets but this requires a specialised imaging sequence which tracks the motion of the mitral valve annulus during the cardiac cycle [31]. In the absence of other regurgitant lesions, MRV can also be calculated by subtracting the right ventricle stroke volume (RVSV) from the LVSV i.e. MRV = LVSV – RVSV, using established techniques [28]. However, the calculation of RVSV is less reproducible compared to LVSV due to the extensive trabeculation of the right ventricle (RV). Moreover, associated tricuspid regurgitation is reported in up to 50% of patients with significant mitral regurgitation and this invalidates the use of RVSV to determine MRV [32]. The American College of Cardiology [2,8] has established echocardiographic criteria for grading the severity of mitral regurgitation. In the absence of established criteria for MRI, the findings of this study, derived from LV volume and ascending aortic flow measurements, can be noted: mild = RF ≤ 15%, moderate = RF 16–24%, moderate-severe = RF 25–42%, severe = RF > 42%.

A further feature of severe mitral regurgitation is reversal of flow in the pulmonary veins during LV systole, which may be visible in the 4 chamber and certain mitral stack cines [9].

Evaluation of mitral valve dysfunction from standard, routinely acquired MRI imaging planes alone is rarely adequate. The proposed technique by Kim RJ et al [9], with additional imaging of the mitral valve based on its anatomy, allows more detailed evaluation of its dysfunction.

In degenerative valve disease, MRI allows determination of the leaflet scallop, responsible for the valve dysfunction e.g. P2 or P3 prolapse, and hence helps guide surgical repair. In rheumatic valve disease, ÌRI allows assessment of the severity of valve restriction and hence helps determine the feasibility of valve repair and the need for valve replacement. In functional mitral regurgitation due to ischemic heart disease or cardiomyopathy, it confirms the diagnosis and helps exclude coexisting degenerative valve disease. Comparison of the accuracy and reproducibility of MRI using this technique with echocardiography, especially transesophageal echocardiography, and findings at surgery will need to be done. Two recent studies using similar techniques as described here, but without the additional slices taken at the commissural ends of the mitral valve, have recently been published [33,34]. The first study reported a sensitivity and specificity of 89% and 88% respectively for detecting flail or prolapsed leaflets compared to findings at surgery in 47 patients. This compared with a sensitivity and specificity of 93% and 88% respectively for transesophageal echocardiography [33]. The second study reported agreement between CMR assessment and transthoracic echo determination of prolapsed or flail leaflets in 92% of 27 patients [33]. According to the lattest study, there was an excellent concordance between MRI and transthoracic echocardiography in the identification of jet direction and leaflet abnormality. MRI mapping of the mitral valve using a simple protocol can reliably acquire long axis images through the valve, facilitating localisation of leaflet abnormalities and regurgitant jet direction. When compared to modern TTE, the MRI mapping protocol accurately identified the abnormal leaflet in 98% of cases [34]. The difference between the 2 techniques was differentiating leaflet flail from prolapse in 3 patients and MRI failing to detect a borderline prolapse (2 mm) involving an anterior mitral valve leaflet. Using either technique, variation in defining the border between adjacent leaflet segments (e.g. A1 from A2) can lead to minor differences in classification but is less likely to effect the decision for valve repair versus replacement. The presence of a flail mitral valve leaflet identifies patients who are at a higher risk of sudden cardiac death and may warrant early surgery if the valve is repairable [35]. The discrepancies in classification of prolapse and flail segments may also in part be due to superior spatial resolution of echocardiographic over MRI when there are adequate echocardiographic windows. MRI spatial resolution is dependent on the voxel size and the slice thickness of the planes used. Hence, visualising the direction of the mitral valve leaflet tip (1 – 5 mm thickness dependent on the degree of mitral leaflet thickening) to define segment prolapse versus flail may be difficult. In addition, insufficient contrast between the signal loss defining the origin of the regurgitant jet and the distal mitral leaflet tip may contribute to the minor differences seen. An advantage of MRI compared to TTE is that because there is no limitation of imaging windows the MRI mapping protocol enabled a complete and systematic assessment of the mitral valve in every patient. Acquisition of the mapping images is usually efficient, requiring on average 7 cine images, and between 5 to 10 minutes per patient. MRI is an accurate, reproducible, and non-invasive manner, potentially enabling better estimation of the timing and type of surgical intervention. Cine MRI is a highly sensitive diagnostic tool to assess changes in LV mass and volume [36-39]. This is supported by Bottini et al. They showed that MRI is the most precise method for measuring LV mass when compared to Transthoracic echocardiography in hypertensive patients [40], suggesting that for more specific questions MRI may be the more reliable imaging tool. Francois et al. have shown that MRI assessment of LV mass correlated well with true LV mass measurements during autopsy [41]. However, MRI is expensive, time-consuming, and only available in specialized centers and therefore no alternative for routine patient follow-up in smaller hospitals and private practices.

## Conclusion

In patients with mitral valve regurgitation MRI has an established role in the assessment of LV size and function and mitral regurgitation severity. With the addition of mitral valve mapping, MRI can potentially provide a comprehensive assessment of mitral regurgitation. Comprehensive assessment of mitral regurgitation requires assessment of: (a) its severity to determine the need for surgical intervention, (b) the mechanism of the dysfunction to determine the type of surgical intervention required (leaflet prolapse/restriction, including the leaflet scallops involved: A1-P1, A2-P2, A3-P3); (c) LV volumes and function to determine the timing and risks of surgery; and, in ischemic mitral regurgitation, (d) LV viability. Such comprehensive assessment is feasible in a single MRI examination but needs a defined protocol, as described in this paper. When used optimally, MRI can complement existing imaging modalities such as echocardiography in the assessment of patients with mitral regurgitation. The fixed imaging planes of MRI and its suboptimal through-plane resolution rarely permit adequate visualisation of the chordal structures to identify rupture or elongation accurately. MRI is also not suited for visualisation of leaflet and annular calcification which are important factors influencing the probability of successful valve repair. MRI, when used optimally, may therefore play a useful role in assessing the mitral valve in patients in whom transthoracic echocardiography has not provided adequate imaging and in whom transesophageal echocardiography is considered too invasive.

## Competing interests

The authors declare that they have no competing interests.

## Authors' contributions

All authors: 1. have made substantial contributions to conception and design, or acquisition of data, or analysis and interpretation of data; 2. have been involved in drafting the manuscript or revisiting it critically for important intellectual content; 3. have given final approval of the version to be published.
